# Immunotoxicity assessment of CdSe/ZnS quantum dots in macrophages, lymphocytes and BALB/c mice

**DOI:** 10.1186/s12951-016-0162-4

**Published:** 2016-02-04

**Authors:** Xiaomei Wang, Jinglin Tian, Ken-Tye Yong, Xuedan Zhu, Marie Chia-Mi Lin, Wenxiao Jiang, Jiefeng Li, Qijun Huang, Guimiao Lin

**Affiliations:** College of Life Science and Oceanography, Shenzhen University, Shenzhen, 518060 People’s Republic of China; Key Lab of Biomedical Engineering, School of Medicine, Shenzhen University, Shenzhen, 518060 People’s Republic of China; School of Electrical and Electronic Engineering, Nanyang Technological University, Singapore, 639798 Singapore; The Institute of Urinary and Reproductive, Shenzhen Key lab of Translational Medicine of Tumor, The Engineering Lab of Synthetic Biology, School of Medicine, Shenzhen University, Shenzhen, 518060 People’s Republic of China

**Keywords:** Quantum dots, Macrophages, Lymphocytes, Cytotoxicity, Immunotoxicity

## Abstract

**Background:**

The toxicity of CdSe/ZnS quantum dots (QDs) in the environment and biological systems has become a major concern for the nanoparticle community. However, the potential toxicity of QDs on immune cells and its corresponding immune functions remains poorly understood. In this study, we investigated the immunotoxicity of CdSe/ZnS QDs using the in vitro in macrophages and lymphocytes and in vivo in BALB/c mice.

**Results:**

Our results indicated that macrophages treated with 1.25 or 2.5 nM QDs exhibited decreased cell viability, increased levels of reactive oxygen species (ROS), elevated apoptotic events, altered phagocytic ability, and decreased release of TNF-α and IL-6 by upon subsequent stimulation with Lipopolysaccharide (LPS). In contrast, lymphocytes exposed to QDs exhibited enhanced cell viability, increased release of TNF-α and IL-6 following exposure with CpG-ODN, and decreased transformation ability treatment in response to LPS. To study the in vivo effects in mice, we showed that QDs injection did not cause significant changes to body weight, hematology, organ histology, and phagocytic function of peritoneal macrophages in QDs-treated mice. In addition, the QDs formulation accumulated in major immune organs for more than 42 days. Lymphocytes from QDs-treated mice showed reduced cell viability, changed subtype proportions, increased TNF-α and IL-6 release, and reduced transformation ability in response to LPS.

**Conclusions:**

Taken together, these results suggested that exposures to CdSe/ZnS QDs could suppress immune-defense against foreign stimuli, which in turn could result in increased susceptibility of hosts to diseases.

**Electronic supplementary material:**

The online version of this article (doi:10.1186/s12951-016-0162-4) contains supplementary material, which is available to authorized users.

## Background

Quantum dots (QDs) are fluorescent semiconductor nanocrystals made of semiconductor materials such as CdTe or CdSe [1]. Many unique optical features of QDs make them useful for applications in biological and medical fields. For example, they have excellent photoluminescence (PL) quantum yield, high photostability, broad absorption band with narrow emission spectra, and size-tunable fluorescent peaks. Over the years, with increasingly large scale production and application, the likelihood of human exposing to QDs is highly possible. In particular, these nanocrystals have been found from environments as the degradation products. To date, the potential risks of QDs on the human health are largely unknown, as our knowledge of the in vitro and in vivo toxicities remain elusive.

Recently published reports have provided limited information about the toxicity of QDs to cells, and most toxicological assessments were performed using in vitro and in vivo models [2–4]. The in vitro toxicities have been assessed using a variety of cell models [5, 6]. However, the collected data is inconclusive owing to the intrinsic properties of QDs such as chemical components, size and surface modification [7, 8]. Moreover, little is known about the molecular mechanisms of the QDs-induced toxicity effects on immune cells [9]. In comparison to in vitro investigations, there has been little information about QDs-induced in vivo toxicity [10, 11]. Previous studies have shown that QDs are accumulated in the spleen (which is one of important immune organs) when administrated through tail vein injection [3]. However, the consequent biological effects on immune functions were rarely addressed on the QDs exposure or accumulation in the reticuloendothelial organs.

The immune system within an organism is a complicated system of many biological components and processes. Both macrophages and lymphocytes (e.g. T lymphocytes, B lymphocytes and natural killer cells) are important immune cells which mediated the innate and adapted immunity of human body. Previously, we have reported that carboxyl-terminated CdSe/ZnS QDs inhibited the cell proliferation of macrophages and the data showed that CdSe/ZnS QDs treatments indeed affect the cell viability of the macrophages [12]. But the molecular mechanisms responsible for QDs-mediated cytotoxicity remain unknown. It is also not known whether QDs influence the immune function of macrophages or other immune cells toward immune stimulus such as bacterial endotoxin and viral nucleic acids. So far, only handful of reports about cytotoxicity of immune cells pertaining to QDs is available in the literature [9] and the potential risk of QDs in vivo in immune functions remains unexplored.

In this study, we used in vitro and in vivo screening strategies to evaluate the immunotoxicity of QDs exposures. The current study employed models of macrophages and spleen-derived lymphocytes, which were previously used to evaluate the immune toxicity or response [4, 13, 14]. Basically, the cell uptake, viability, apoptosis and reactive oxygen species (ROS) generation were studied. And further studies were performed to test the effects of QDs on these cells in subsequent exposures to foreign substances including lipopolysaccharides (LPS) and CpG oligodeoxynucleotides (CpG-ODN) which are well-characterized immune stimuli recognizing different types of Toll-like receptors [15, 16]. For in vivo assay, the biodistribution, hematology, organ histology, proportion and functions of spleen derived lymphocyte, as well as phagocytic capacity of peritoneal macrophages from treated mice were determined at a relatively high concentration (0.41 mg/kg of Cd). To our knowledge, this is first study to demonstrate QDs-induced immunologic function impairment in vitro in immune cells and in vivo in BALB/c mice.

## Methods

### Preparation and characterization of QDs

ZnS-coated CdSe QDs (CdSe/ZnS, Q21321MP) were purchased from Invitrogen Company. This core–shell material is coated with a polymer layer and it is terminated with COOH surface groups. The photoluminescence emission spectra of CdSe/ZnS were measured by a Fluorescence spectrophotometer (Cary Eclipse, Agilent, USA) with an excitation wavelength of 400 nm. UV–Visible-NIR absorption spectra were collected using a UV–Vis-NIR spectrophotometer (Cary 5000, Agilent, USA). The hydrodynamic size distribution of the CdSe/ZnS was obtained by a dynamic light scattering (DLS) machine (Zetasizer Nano ZS, Malvern, UK). The morphology of the CdSe/ZnS quantum dots was obtained with an FEI Tecnai G2 F20 S-TWIN transmission electron microscopy (TEM) operating at an accelerating voltage of 200 kV at room temperature.

### Cell culture

Macrophage cell line RAW264.7 was obtained from American Type Culture Collection (ATCC) and cultured in Dulbecco’s modified Eagle’s medium (DMEM, Gibco, USA) supplemented with 10 % fetal bovine serum (FBS, Gibco, USA) and 100 U/mL penicillin/streptomycin (Gibco, USA). Mouse spleen lymphocytes were isolated from BALB/c mice and grown in RPMI 1640 medium with 10 % FBS. All cells were cultured at 37 °C in humidified 5 % CO_2_ atmosphere.

### Laser scanning confocal imaging

The day before experiment, RAW264.7 macrophages (5.0 × 10^5^/dish) were seeded in 3.5 cm dishes. Macrophages were left untreated or exposed to 1.25 and 2.5 nM CdSe/ZnS QDs for 24 or 48 h. Then the cells were fixed, stained with DAPI, and imaged according to the procedure published previously [12].

### Cell viability detected by WST assay

The cell viability of macrophages was evaluated by WST assay using Cell Counting Kit-8 kit (Dojindo, Japan). The day before assay, macrophages (1.0 × 10^4^ cells/well) or spleen lymphocytes (2.5 × 10^5^ cells/well) were seeded in 96-well plates. Cells were left untreated or treated with 1.25 or 2.5 nM CdSe/ZnS QDs and incubated for 24 or 48 h respectively. The CCK-8 assay solution (10 μL/well) was added and cells were incubated for 3 h. Absorbance was measured with a microplate reader (Multiskan FC, ThermoFisher, Finland) at a wavelength of 450 nm. The cell viability was calculated by normalizing the absorbance of the sample well against that of the control well and expressed as a percentage, assigning the cell viability of non-treated cells as 100 %.

### Cell apoptosis detection by Western blot analysis

The day before assay, RAW264.7 macrophages (5.0 × 10^5^ cells/well) were seeded in 6-well plates. Cells were left untreated or exposed to 1.25, 2.5 nM CdSe/ZnS QDs for 48 h. The medium was removed and cells were washed three times with PBS. Protein was extracted by the cold mammalian protein extraction reagent (Thermo, USA) according to the manufacturer’s instructions. A total of 15 μg proteins were resolved by 12 % SDS-PAGE and transferred to 0.22 μm polyvinylidene difluoride membranes (Millipore, USA). Membranes were soaked in blocking buffer (Skim Milk, China) for 1 h at room temperature and incubated with rabbit antibodies of GAPDH (1:1000, ABclonal, USA), Caspase-9 (1:1000, Proteintech, China), Caspase-3 (1:1000, Proteintech, China) at 4 °C overnight. Membranes were washed with TBST (10 mM Tris–HCL, 150 mM NaCl, and 0.05 % Tween 20) three times at 10 min interval. Membranes were incubated with goat anti-rabbit secondary antibodies (1:1000, ABclonal, USA) for 1 h. Then the membranes were washed three times with TBST. Protein bands were visualized with clarity western ECL substrate (BioRad, USA) and the pictures were obtained by chemiscope western blot imaging system (Clinx science Instruments, China).

### Detection of reactive oxygen species (ROS)

The production of cellular ROS was detected by the carboxy-dichlorofluorescein diacetate (carboxy-DCFH-DA) assay. Briefly, 10 μM 2′-7′-Dichlorodihydrofluorescein diacetate (DCFH-DA) (Sigma-Aldrich, USA) was added to each well of 6-well plates for 4 h exposures. At the same time, cells were left untreated or treated with CdSe/ZnS QDs at 1.25, 2.5 nM respectively and cultured for 4 h. Then the following assay was performed according to the manufacturer’s instructions. The signals of dichlorodihydrofluorescein (DCF) fluorescence were analyzed using a flow cytometry (FACS Calibur, BD, USA).

### Measurement of cytokine production

RAW264.7 macrophages (1.0 × 10^4^ cells/well) or spleen lymphocytes from normal mice (5 × 10^5^ cells/well) were seeded in 96-well plates and cultured overnight. The cells were left untreated or treated with 1.25, 2.5 nM of CdSe/ZnS QDs respectively and incubated for 6 h. The cell medium was replaced and cells were stimulated with 8 ng/mL LPS or 10 μg/mL CpG-ODN (5′-TCCATGAGTTCCTGACGTT-3′) respectively for 24 h (37 °C, 5 % CO_2_). The levels of TNF-α and IL-6 in culture supernatants were measured using enzyme-linked immunosorbent assay (ELISA) (eBioscience, USA), according to the manufacturer’s instructions.

### Neutral red uptake assay

The phagocytic capacity of macrophages was detected by neutral red uptake assay (Beyotime, China). The cells (1.0 × 10^4^ per well) were seeded in 96-well plate and incubated overnight. The cells were treated with 1.25 or 2.5 nM CdSe/ZnS QDs for 2, 4, 6 and 24 h respectively. After that, 20 μL neutral red solutions were added into each well and the cells were incubated for 2 h. The medium was removed and the cells were washed with PBS once. Then 200 μL of lysis buffer was added into each well and the wells were incubated at room temperature for 10 min with constant slight shaking. The absorption of the wells was read at a wavelength of 540 nm by a microplate reader (Multiskan FC, ThermoFisher, Finland) and the uptake efficiency was calculated by normalizing the absorbance of the sample well against that of the control well and expressed as a percentage, assigning the phagocytic ability of non-treated cells as 100 %.

### Transformation assay

Spleen lymphocytes from normal mice were seeded in 96-well plates (2.5 × 10^5^/well) and exposed to 1.25 or 2.5 nM CdSe/ZnS QDs and incubated for 6 h. Lymphocytes from untreated mice were set as control. The cells were subsequently stimulated with 1.25 μg/mL LPS or 8 μg/mL ConA (Concanavalin A) for 48 and 72 h. Then 10 μL CCK-8 solutions was added and incubated for 4 h. Absorbance was measured with a microplate reader (Multiskan FC, ThermoFisher, Finland) at a wavelength of 450 nm. The lymphocyte transformation percentage was calculated according to the following formulas: (OD_after stimulation−_OD _before stimulation_)/QD _before stimulation_ × 100.

### Animals, blood test, biodistribution analysis and H and E staining

BALB/c male mice of 6-week-old were obtained from Laboratory Animal Center of Guangdong Province and housed in individual ventilation cages in a 12/12 h light/dark cycle. The animal experiments were carried out in accordance with recommendations cited in the Guide for the Care and Use of Laboratory Animals of Laboratory Animals Center of Shenzhen University. And the project and protocols were approved by the Experimental Animal Ethics Committee of Shenzhen University (Permit no.201412012). The mice were injected with 200 μL of PBS or buffered QDs dispersion (2 nmol/kg) by tail vein. After injection, body weight of mice was measured on Day 1, 3, 7, 14, 30, 42 respectively. On Day 1 and Day 42 after the injection, mice of each group were sacrificed. Blood samples were harvested and blood routine examination was performed using the whole blood by a routine blood test instrument (RJ-0C107223, Mindray, China). The fresh major organs were dissected, weighed and organ index was calculated (organ weight/body weight). The tissues were selected for frozen section examination and H and E (haematoxylin and eosin) staining. Frozen section was prepared using a freezing microtome (CM3050S, Leica, Germany) and imaged by a fluorescent microscope (BX51, Olympus, Japan). The amounts of heavy metal elements in the blood and tissue were determined by inductively coupled plasma mass spectrometry (ICP-MS) (7500C1, Agilent, USA) analysis according to the protocol previously published [10].

### Analysis of spleen-derived lymphocytes from treated mice

The mice were injected with 200 μL of PBS or buffered QDs dispersion by tail vein. 42 days later, the mice were sacrificed. The spleen was dissected and total lymphocytes were harvested for cell viability assay, subset analysis, cytokine release analysis and transformation assay. For analysis of subset, 1 × 10^6^ spleen lymphocytes were collected and washed with cell staining buffer (Biolegend, USA) once, resuspended with 100 μL staining buffer, and stained with anti-CD3ɛ ^FITC^ (1 μg), anti-CD19 ^PE^ (0.25 μg) and anti-CD49b ^PerCP^ (0.25 μg) for 20 min. The supernant was removed after centrifugation of 1000 rpm for 5 min. Then the cells were washed with staining buffer once, resuspended in 500 μL PBS buffer and immediately analyzed by flow cytometry (FACS Calibur, BD, USA). Cell viability assay, cytokine release analysis and transformation assay were performed according to the methods described above.

### Statistical analysis

All experimental data were presented as the mean ± standard deviation (SD). T test was employed to assess the mean of the two groups and the mean difference among the groups compared to control by analysis of variance (ANOVA). P < 0.05 was considered to indicate a statistically significant difference.

## Results

### Characterization of the CdSe/ZnS QDs

**Fig. 1:**
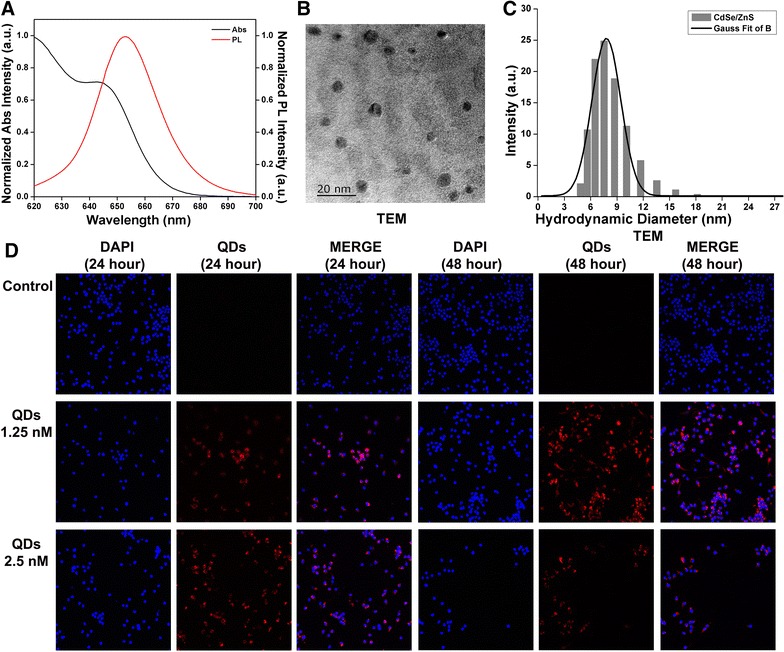
Characterization of CdSe/ZnS QDs and uptake of CdSe/ZnS QDs by macrophages. **A** The photoluminescence emission and absorption spectra. **B** TEM image of CdSe/ZnS QDs. **C** The hydrodynamic size distribution of the CdSe/ZnS QDs. **D** Fluorescent images of RAW264.7 macrophages treated with CdSe/ZnS QDs for 24 and 48 h. Left, middle and right panels are DAPI signal, QDs signal and merged images, respectively. The cell nucleus was stained with DAPI (*pseudo-colored in blue*) and the signals from the QDs were in *red*

We first showed that the maximum emission for the CdSe/ZnS is 655 nm from the florescence spectra (Fig. 1A). Figure 1B showed the TEM image of CdSe/ZnS QDs. The average hydrodynamic diameter of CdSe/ZnS was around 8 nm (Fig. 1C). When the CdSe/ZnS QDs were kept at 4 °C, no aggregation or precipitation was observed during the period of storage, suggesting that the CdSe/ZnS QDs nanocrystals are highly monodispersed.

### Uptake of the CdSe/ZnS QDs by immune cells

As shown in Fig. 1D, the confocal images of CdSe/ZnS QDs treated mouse macrophages indicated that the fluorescent signal was detectable even after 24 or 48 h of treatment. These observations suggest that the CdSe/ZnS QDs can be taken up by macrophages and stayed in cells continuously for over 48 h without being digested or discharged. As the adherent ability of lymphocytes is poor and cells are easy to detach from the cultured dishes, we determined the uptake of CdSe/ZnS QDs by lymphocytes using flowcytometry assay instead of confocal imaging. As shown in Additional file 1: Figure S1, lymphocytes treated with CdSe/ZnS QDs had negative fluorescent signal, suggesting that the QDs can’t go into the lymphocytes.

### Effect of in vitro exposure to QDs on macrophages

**Fig. 2:**
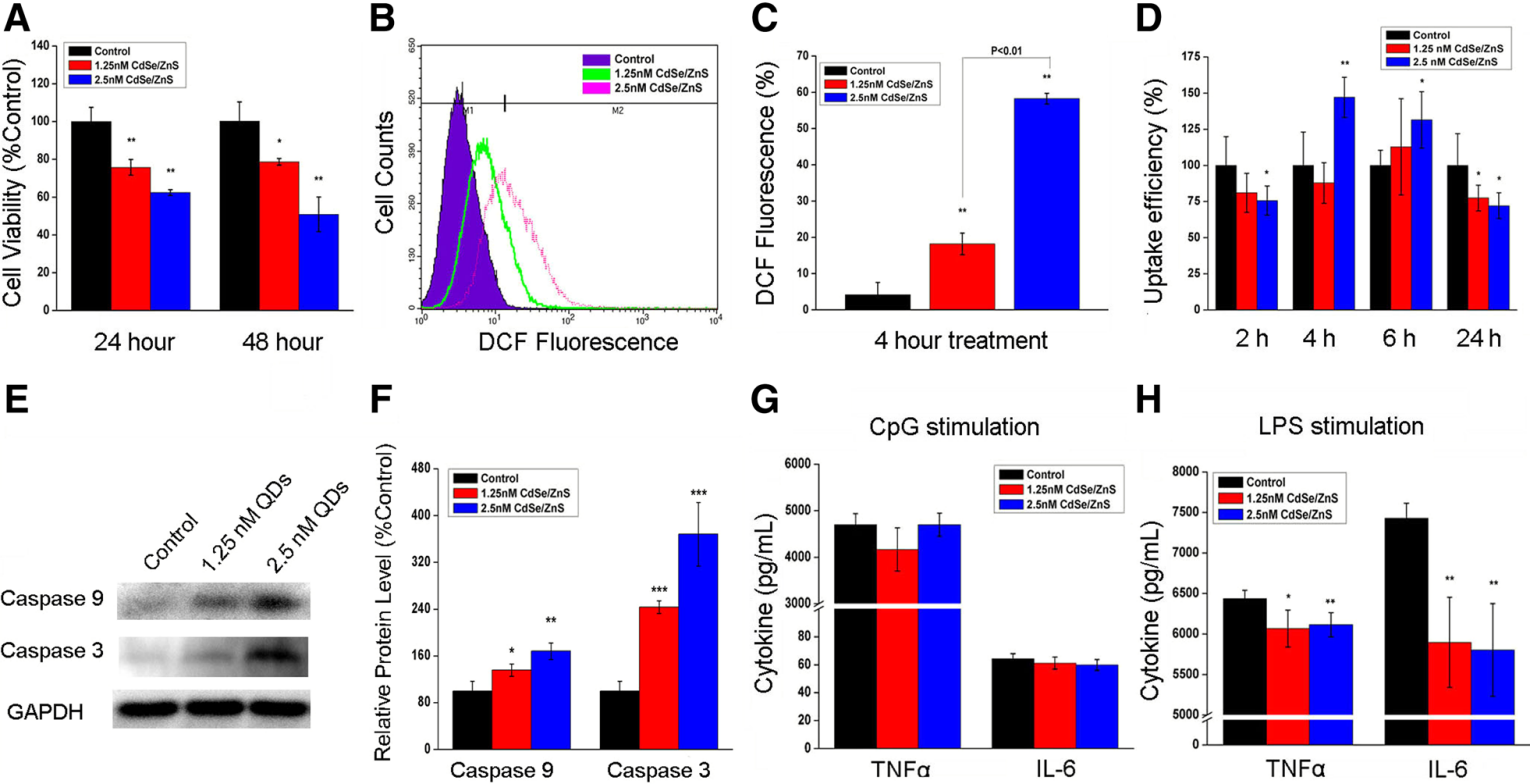
Effect of in vitro exposure to QDs on macrophages. **A** Cell viabilities of RAW264.7 macrophages treated with QDs (n = 5). **B** Flow cytometry analysis of DCF fluorescence reflecting intracellular ROS level in RAW264.7 macrophages treated with QDs for 4 h. **C** Statistical analysis of ROS level in RAW264.7 macrophages (n = 3). **D** *Neutral red* uptake ability of RAW264.7 macrophages after exposure to QDs for 2, 4, 6 and 24 h (n = 6). **E** Representative pictures of Western blot analysis. **F** Densitometric analysis of protein expression levels of cleaved Caspase 3 and Caspase 9. (*P < 0.05, **P < 0.01, ***P < 0.001 vs. control). **G** and **H** The levels of TNF-α or IL-6 released by RAW264.7 macrophages after re-stimulation with CpG or LPS (n = 6). All values are mean ± SD, *P < 0.05 vs. control, **P < 0.01 vs. control

As shown in Fig. 2A, the cell viability of macrophages treated with CdSe/ZnS QDs for 24 or 48 h was significantly lower than that of the untreated cells. In addition, the cell viability of macrophages treated with three other types of QDs was also detected by MTT assay and the results showed the same trend (Additional file 1: Figure S4). To probe the mechanism of cytotoxicity, the production of cellular ROS and apoptotic events were examined. Figure 2B, C showed that treating macrophages with 1.25 and 2.5 nM QDs for 4 h resulted in significant increase in intracellular ROS level. Figure 2E, F showed the protein expression levels of Caspase 9 and Caspase 3 which are involved in the activation cascade of Caspases responsible for apoptosis execution. Our data showed that the expression of cleaved Caspase 3 and Caspase 9 significantly increased in the RAW264.7 cells treated with CdSe/ZnS QDs, suggesting increased apoptotic events occurred in the cells. Phagocytosis is another important function of macrophages. As shown in Fig. 2D, 1.25 nM QDs treatment reduced the phagocytic activity at 24 h post-treatment. When treated with 2.5 nM QDs, a significant decrease in phagocytic activity was observed at 2 or 24 h post-treatment, but surprisingly, an increase in phagocytic ability were observed at 4 and 6 h after treatment. These results suggested that QDs treatment caused immune dysfunction in macrophages.

In addition, we also exposed macrophages first with CdSe/ZnS QDs for 6 h and then stimulated with LPS and CpG-ODN, which are well known immune stimuli recognizing different types of Toll-like receptors. As shown in Fig. 2G, macrophages treated with 1.25 or 2.5 nM QDs showed no change in the release of IL-6 and TNF-α after exposure to CpG-ODN. However, significantly lower amounts of IL-6 and TNF-α secretion were observed when stimulated with LPS (Fig. 2H).

### Effect of in vitro exposure to QDs on spleen-derived lymphocytes

**Fig. 3:**
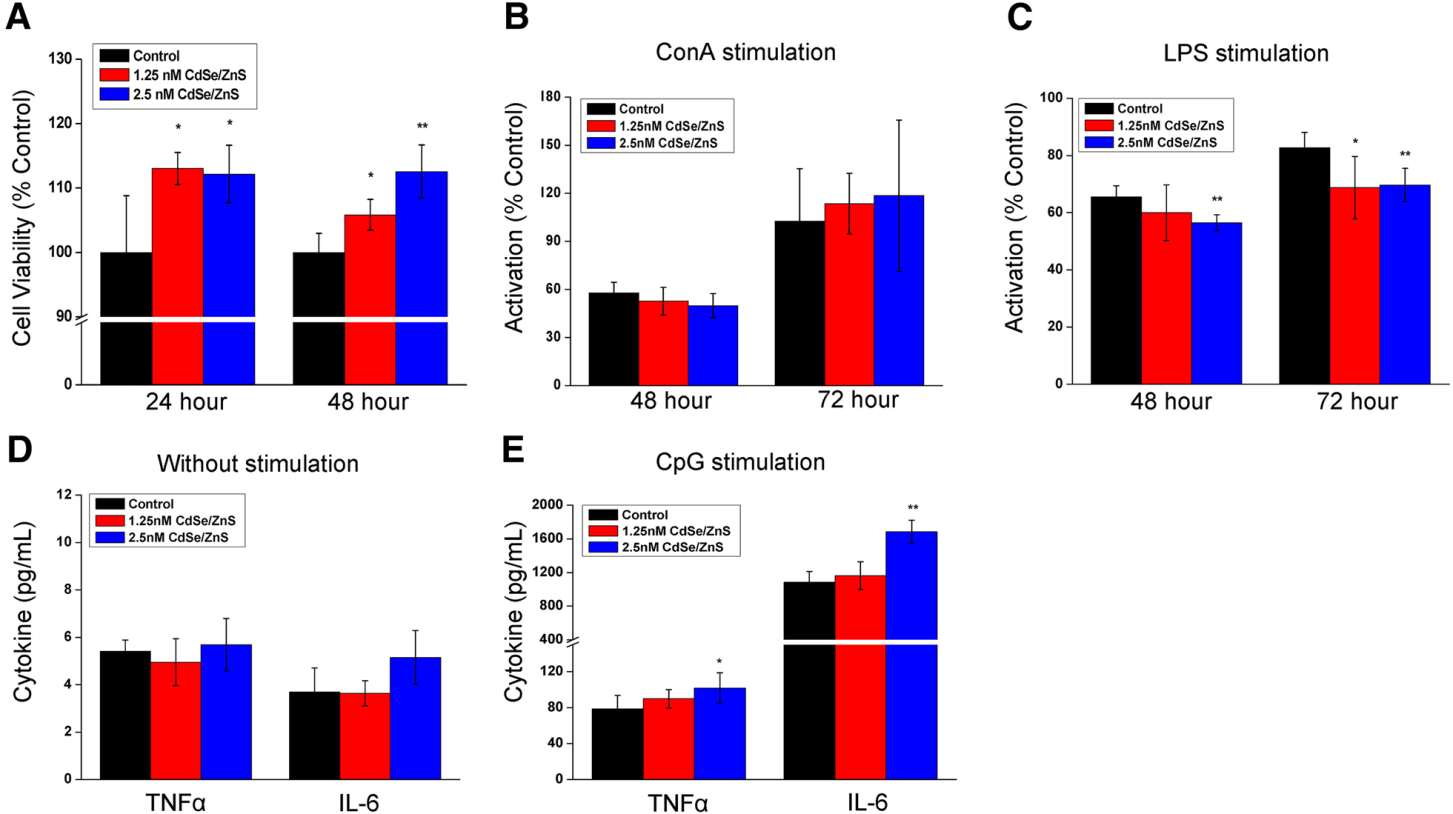
Effect of in vitro exposure to QDs on spleen-derived lymphocytes. **A** Cell viabilities of lymphocytes treated with QDs for 24 or 48 h (n = 5). **B** and **C** Activation abilities of lymphocytes treated with QDs in response to ConA or LPS (n = 6). **D**, **E** The levels of TNF-α or IL-6 released by QDs-pretreated lymphocytes after re-stimulation with PBS or CpG (n = 6). All the values are the mean ± SD, *P < 0.05 vs. control, **P < 0.01 vs. control

In lymphocytes, CdSe/ZnS QDs exposure alone led to enhanced cell viability (Fig. 3A). We further investigated the effect of CdSe/ZnS QDs on transformation ability of lymphocytes after subsequent exposure to ConA and LPS. As we know, when lymphocytes are activated, the cells will transform into lymphocytoblasts with increased volume and metabolic activity. As shown in Fig. 3B, after subsequent exposure to ConA for 48 or 72 h, lymphocytes treated with 1.25 and 2.5 nM CdSe/ZnS QDs showed no significant change in transformation abilities as compared to untreated cells. However, when the lymphocytes were subsequently stimulated by LPS, CdSe/ZnS QDs treatment at 1.25 nM caused no observed change in transformation ability at 48 h, but resulted in obvious decrease at 72 h (Fig. 3C). In addition, when the dosage of QDs reached 2.5 nM, the activation abilities of lymphocytes towards LPS were obviously inhibited at both 48 and 72-hour time points. Figure 3D, CdSe/ZnS QDs exposure alone caused no significant change in the release of IL-6 and TNF-α. When lymphocytes were subsequently stimulated by CpG-ODN, the levels of cytokines released by lymphocytes remained unchanged at the dose of 1.25 nM, but increased at the dose of 2.5 nM (Fig. 3E).

### In vivo distribution of QDs in the blood and major immune organs

**Fig. 4:**
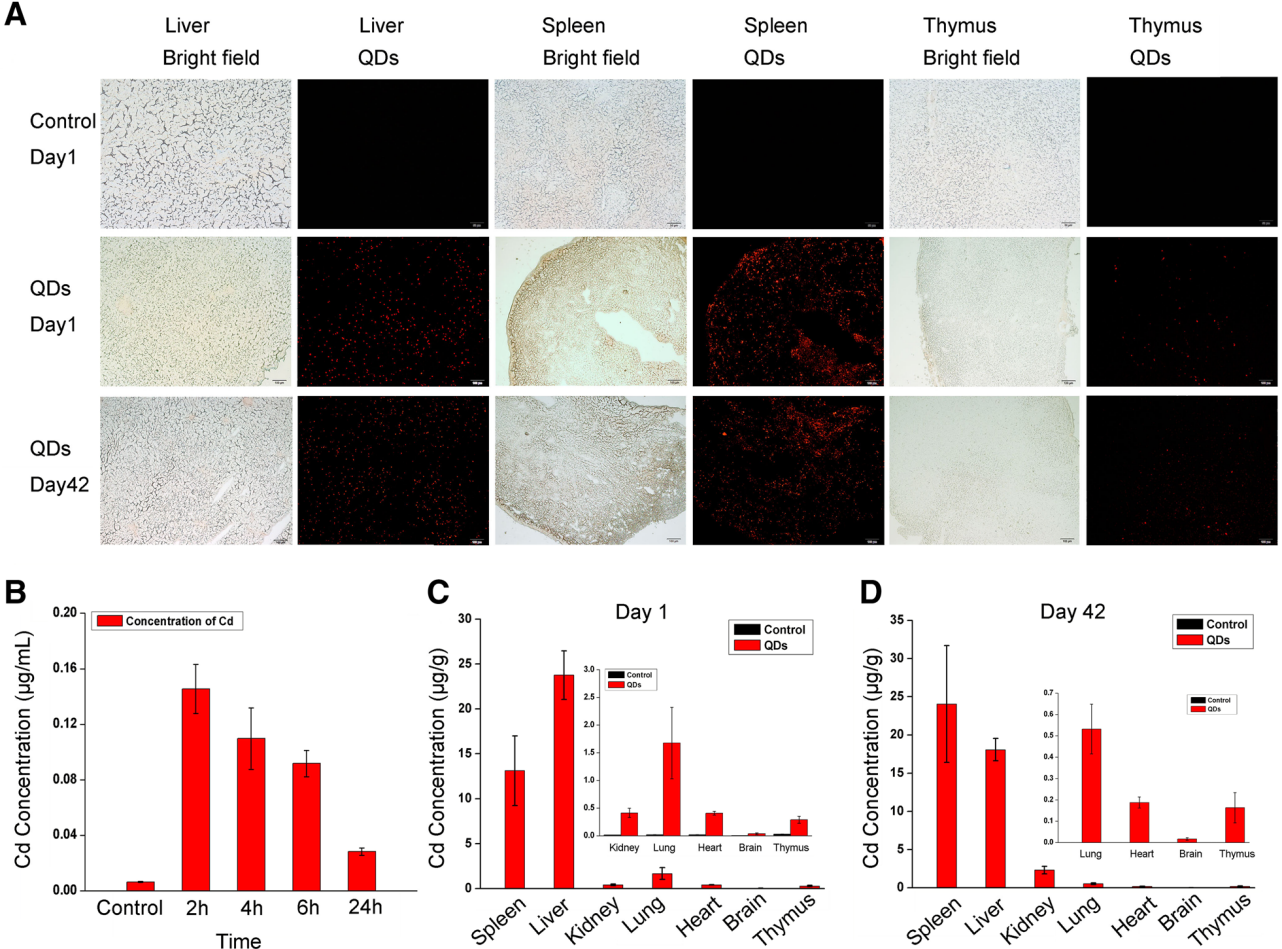
In vivo distribution of QDs in blood and major immune organs. **A** *Fluorescence* images of liver, spleen and thymus of the mice emitted by QDs on Day 1 and Day 42 after injection. **B** ICP-MS analysis of Cd concentrations in blood at 2, 4, 6 and 24 h after injection. **C** and **D** ICP-MS analysis of Cd concentrations in major organs from QDs-treated mice on Day 1 and Day 42 after injection

To evaluate the immunotoxicity of QDs in vivo, CdSe/ZnS QDs were administered into the mice intravenously. In order to assess the dose of CdSe/ZnS QDs formulation, we first performed ICP-MS analysis to calculate the concentrations of Cd, Se and Zn. Our results indicated that the CdSe/ZnS QDs formulation we injected corresponded to 0.41 mg/kg of Cd, 0.012 mg/kg of Se, and 0.0364 mg/kg of Zn. On Day 1 and 42 after injection, mice were sacrificed; their major organs were dissected for fluorescence imaging. Our results showed that strong fluorescence signals were observed in the spleen and liver even at 42 days post-injection (Fig. 4A). The fluorescence signals were also detectable in thymus. However, little fluorescence signal was detected in the lung, heart, kidney or brain (data not shown). These results manifested that the majority of CdSe/ZnS QDs were taken up by the immune organs and remain intact with sustainable fluorescence emitting ability.

**Fig. 5:**
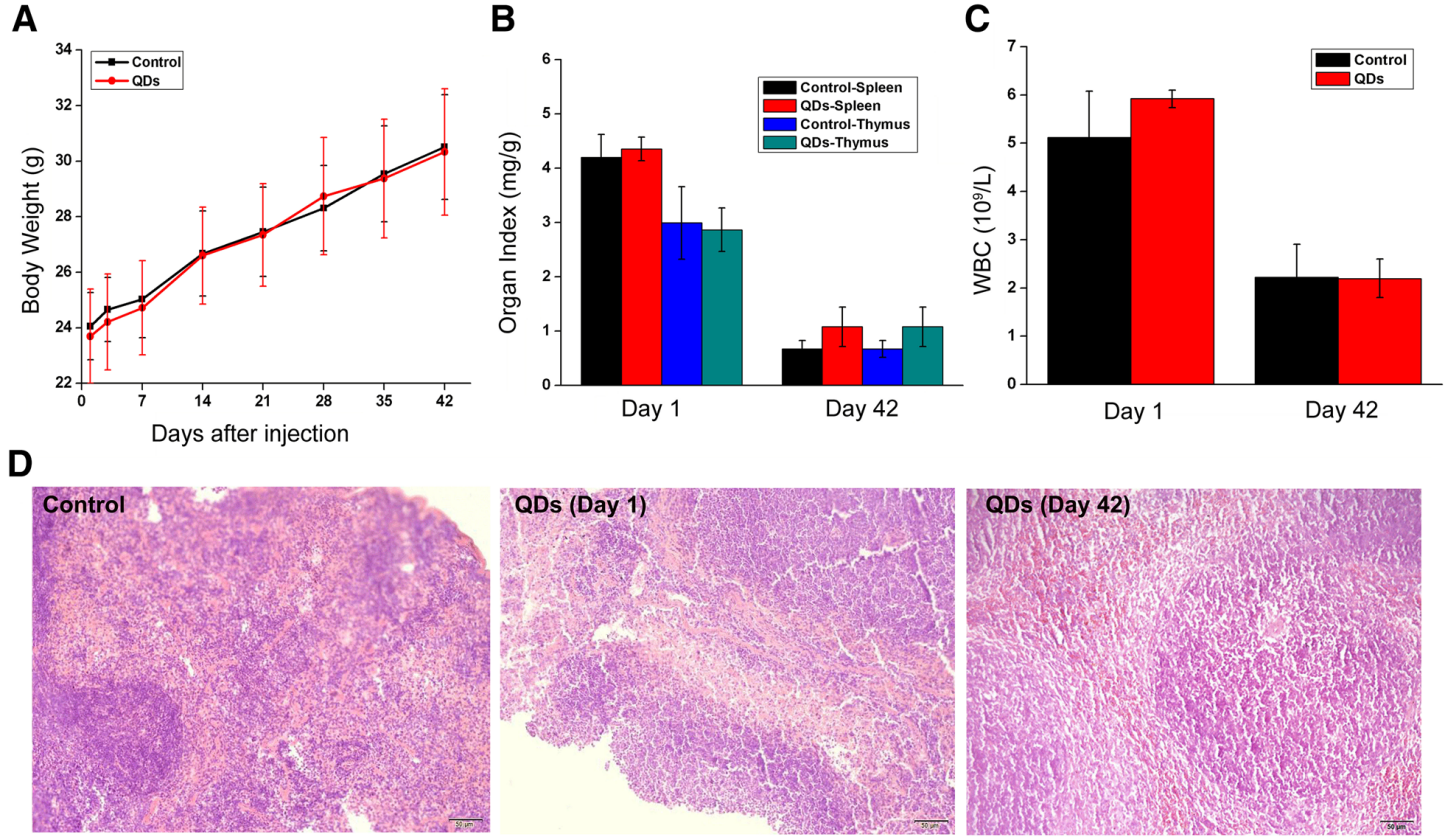
Effects of in vivo exposure to QDs on body weight, WBC quantity, immune organ index and histomorphology. **A** The weights of mice were monitored after injection of CdSe/ZnS QDs/buffer solution through tail vein during 42 days period. **B** Major immune organ index of treated mice. **C** Quantity of white blood cells in blood. **D** HE staining of spleen from QDs-treated or untreated mice

To further quantify the accumulation of cadmium derived from CdSe/ZnS QDs, the concentrations of Cd and Se in the blood and tissues were measured by ICP-MS analysis. Figure 4B showed the Cd concentration in blood circulation at 2 h, 4 h, 6 h and 24 h after injection. After injection, the initial concentration of Cd is approximately 5.46 μg/mL. At 2 and 4 h, the Cd concentration was 0.15 ± 0.022 and 0.11 ± 0.018 μg/mL, respectively, but at 6 and 24 h, the Cd concentration in blood decreased to 0.09 ± 0.009 and 0.028 ± 0.003 μg/mL, respectively. The blood clearance profile revealed that the CdSe/ZnS QDs can be cleared by the blood quickly. Figure 5C–D shows the Cd concentration in major organs on Day 1 and Day 42 after injection. The Cd element was found to accumulate in all the tested organs, including heart, liver, spleen, lung, kidney, brain and thymus on Day 1 and Day42. The Cd concentrations in spleen and thymus were 13.12 ± 3.885 and 0.29 ± 0.065 μg/g on Day 1, respectively. On Day 42, the Cd component still remarkably accumulated in spleen and thymus, where the Cd concentrations were 24.05 ± 7.651 and 0.16 ± 0.071 μg/g, respectively. In addition, we also determined the Se concentration in blood and major organs (Additional file 1: Figure S2), and our data showed that Se element from CdSe/ZnS QDs had predominantly accumulated in the spleen and liver.

### Effects of in vivo exposure to QDs on WBC quantity, immune organ index and histomorphology

To further investigate the immunotoxicity effects and biocompatibility of CdSe/ZnS QDs formulation in vivo, the body weight and immune organ index of mice were monitored, and no significant difference was observed between QDs-treated and untreated animals (Fig. 5A, B). Blood routine examination was performed to determine the quantity of white blood cells (WBC) in blood. The quantity of WBC in blood reflects the overall immunity of the body and assisting in proofing the presence of inflammation. Figure 5C showed that no difference in WBC quantity between these two groups, suggesting no inflammation existed in the mice with CdSe/ZnS QDs exposure. Besides WBC, other major blood biomarkers including red blood cell count (RBC), Hemoglobin (Hb), hematocrit (Hct), Platelet (Plt), mean corpuscular volume (MCV) and mean platelet volume (MPV) were analyzed. Our results showed that all measured factors were within normal ranges (data not shown). In addition, H and E staining was performed to observe the histological changes of spleens from mice at Day 1 and Day 42. Figure 5D showed no pathological injury in the structure of the spleens from treated mice.

### In vivo exposure to QDs caused the dysfunctions of immune cells in vivo in BALB/c mice

**Fig. 6:**
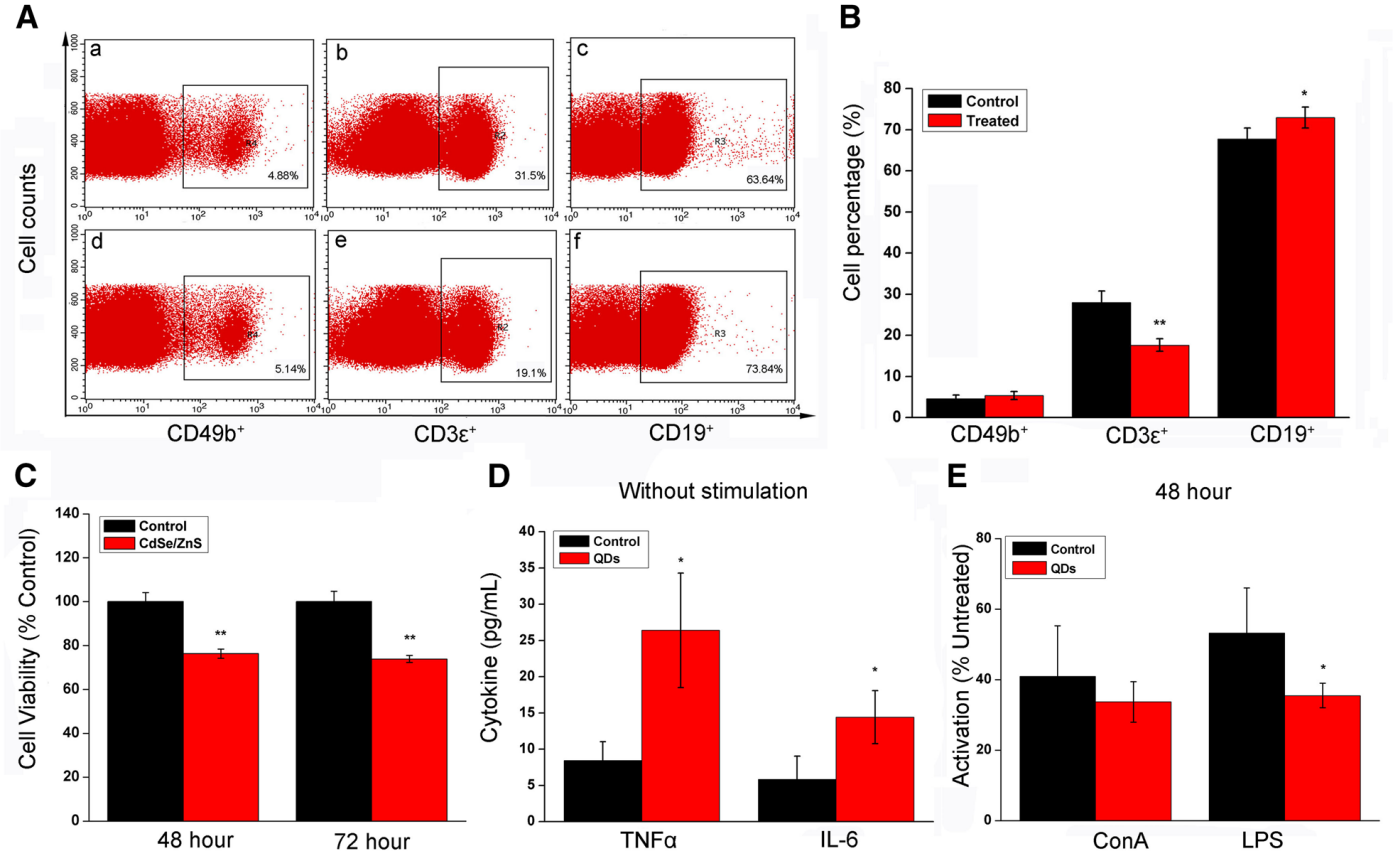
In vivo exposure to QDs caused dysfunctions of immune cells in BALB/c mice. **A** Representative FACS pictures describing the subsets of spleen lymphocytes from untreated or treated mice. *a–c* control; *d–f* QDs. **B** Statistical analysis of T lymphocytes (CD3ɛ^+^), B lymphocytes (CD19^+^) and NK lymphocytes (CD49b^+^) (n = 5). **C** Cell viability of spleen lymphocytes from QDs-treated mice on Day 42 (n = 4). **D** Cytokines release of spleen lymphocytes from QDs-treated or untreated mice (n = 4). **E** Activation ability of lymphocytes from QDs-treated or untreated mice in response to foreign stimulus LPS and ConA for 48 h (n = 4). The values are the mean ± SD, *P < 0.05 vs. control, **P < 0.01 vs. control

To evaluate the in vivo effect of QDs exposure on the immune cells, we first analyzed the subsets of lymphocytes, T lymphocytes (CD3ɛ^+^), B lymphocytes (CD19^+^) and NK lymphocytes (CD49b^+^) from treated mice using flowcytometry. As shown in Fig. 6A, B, exposure to CdSe/ZnS QDs resulted in decreased percentage of CD3ɛ^+^-T lymphocytes, increased percentage of CD19^+^-B lymphocytes, but caused no effects on the proportion of CD49b^+^-NK cells in total lymphocytes from mice. In addition, Fig. 6C showed that CdSe/ZnS QDs exposure led to decreased cell viability of total lymphocytes. To further evaluate the in vivo immune ability of QDs-treated mice, the chicken erythrocytes phagocytosis assay was performed (Additional file 1: Figure S3A). Additional file 1: Figure S3B, S3C showed no significant differences in phagocytic percentage between QDs-treated or PBS-treated mice. In addition, the immune functions of lymphocytes from treated mice were evaluated. Figure 6D, lymphocytes from QDs-treated mice showed increased release of IL-6 and TNF-α as compared to those from PBS-treated mice. In addition, the transformation ability of lymphocytes was determined to analyze immune function of the response to foreign stimulus. As shown in Fig. 6E, CdSe/ZnS QDs treatment resulted in a decrease of transformation ability when lymphocytes were exposed to LPS. These results suggested that the immune functions of lymphocytes from spleen are indeed affected by the CdSe/ZnS QDs exposure.

## Discussions

Previously, we have reported the cytotoxicity effects of commercial carboxyl-terminated CdSe/ZnS QDs on RAW264.7 murine macrophage cells (Lin et al. 2014). Here we extend the study using in vitro immune cell model and in vivo BALB/c mice. In the present work, we investigate the potential molecular mechanism of CdSe/ZnS QDs-induced cytotoxicity and the corresponding effects of CdSe/ZnS QDs on the innate and adapted immunity of immune cells towards exogenous substances.

Cadmium containing QDs such as CdTe and CdSe have been shown to induce toxicity in vitro cell model [17]. Here, our data showed that CdSe/ZnS QDs can be captured by macrophages but not by lymphocytes. It is not surprising because macrophages are specific immune cells with phagocytic ability. In addition, our data showed that CdSe/ZnS QDs exposure led to a decrease in cell viability of macrophages, which is largely consistent with previous studies using different cell lines [8, 18] and immune cells [4]. Conversely, CdSe/ZnS QDs treatment caused enhanced cell viability of lymphocytes. Such phenomena might be resulted from the difference between these two cell types. Previously, Shiohara et al. tested the QDs sensitivity to different cell lines and demonstrated that cytotoxicity was dependent on the cell type exposed [19]. RAW264.7 macrophage cell line is immortalized with rapid proliferation and phagocytic ability, but total lymphocytes are primary cells derived from mouse spleen with limited multiplication capacity but strong transformation ability. In response to an immune stimulus, part of small lymphocytes transform into immature mother cells and go under mitosis, which lead to increase cell density and viability. These data suggested that CdSe/ZnS QDs exposure alone will impair the macrophage cell viability, but lead to lymphocyte transformation.

Previous studies suggested that ROS generation plays a key role in the toxicological profile of nanomaterials [20, 21]. Our data showed an increase of intracellular ROS level in macrophages with 4-h exposure of CdSe/ZnS QDs, which align to some extent with the previous study retaining cadmium-based QDs in other cell types [4]. In fact, for each type of nanoparticles with different component, size, and surface modification, the corresponding mechanisms of nanoparticles-induced ROS generation will be different in many ways [22]. Apoptosis has been described as a major mechanism of cell death induced by nanoparticle-induced oxidative stress [23]. Here our data showed that an increase in apoptosis event in cells with QDs exposure, which is consistent with previous study [4]. Taken together, the results on cell uptake, viability, ROS generation and apoptosis indicate that CdSe/ZnS QDs can enter immune cells with exerting obvious cytotoxicity. However, cell function is another important parameter that might change when the QDs are present within the cells. To date, little toxicological data is available for the examination of the toxicity effects of QDs on macrophages and lymphocytes and the corresponding responses of immune cells towards physiological stimulus.

In this study, we first assessed whether macrophages and lymphocytes, which had been first exposed to CdSe/ZnS QDs, were still able to behave normally in response to LPS and CpG-ODN. LPS, also known as endotoxin, is a large molecule consisting of a lipid and a polysaccharide found in Gram-negative bacteria, and they elicit strong immune responses in animals [15]. CpG-ODN is a short unmethylated single-stranded synthetic DNA molecule that has immunostimulatory properties [16]. Our in vitro data showed the release of cytokines of macrophages in response to CpG-ODN was not significantly affected by CdSe/ZnS QDs, but remarkably suppressed in response to LPS. This inconsistent result is not surprising because LPS and CpG-ODN are different immune stimuli and mediate the immune response by different pathway. Both LPS and CpG-ODN are highly conserved structural motifs known as pathogen-associated microbial patterns (PAMPs), which can be recognized by different Toll-Like Receptors (TLRs) expressed on macrophages. LPS detects TLR4, but unmethylated CpG DNA recognizes TLR9 [24]. Here, our data indicated that CdSe/ZnS QDs pretreatment will influence the LPS-TLR4 pathway activation, but has no effect on the biological activities of TLR9 pathway.

The present study goes further to investigate the influence of CdSe/ZnS QDs on macrophages and lymphocytes functionality. The phagocytic activity of macrophages and the transformation ability of lymphocytes with CdSe/ZnS QDs pre-treatment were determined. Our data showed the phagocytic activity of macrophages was decreased after 2 h CdSe/ZnS QDs treatment, but subsequently enhanced after 4 and 6 h treatment. The influence of CdSe/ZnS QDs on macrophages was discovered to be non-time dependent. Such phenomena might be resulted from the competitive uptake process between the neutral red and QDs because CdSe/ZnS QDs was mainly captured by macrophages within 2 h [12], which resulted in decreased uptake of the neutral red. However, 2 h later, the QDs-treated macrophages were activated, and the uptake ability of them was elevated. In addition, we found a significant inhibited uptake ability of macrophages after 24 h treatment, which is possibly owing to the decreased cell viability as described above. As we known, healthy cells with intact lysosomes will take up more neutral red dye than those dead cells or cells undergoing apoptosis [25]. In addition, our results showed that the transformation ability of CdSe/ZnS QDs pretreated-lymphocytes towards LPS was inhibited. These results are not contradictory when compared to those in response to ConA because LPS and ConA are different immune stimuli, which activate different types of lymphocytes. ConA mainly stimulate T cell proliferation, while LPS mainly stimulate B cell proliferation.

To substantiate the in vitro finding of Cds-induced dysfunction on immune cells, we performed in vivo exposure of CdSe/ZnS QDs in BALB/c mice. QDs are within the size range of viruses and large protein, and consequently they may arouse an inflammatory response. The ICP-MS analysis of blood sample revealed that the  CdSe/ZnS QDs can be cleared from the blood quickly. Once the QDs are cleared from the blood stream, they will migrate to tissues or organs. The spleen and thymus, consisting of B and T lymphocytes and accessory cells including macrophages, are the major immune organs. We found that the fluorescence of  CdSe/ZnS QDs injected into BALB/c can be detected in spleen and thymus for up to 42 days post injection. Consistent with our finding, several studies have reported the similar accumulation pattern of cadmium-based Ads in animal models [11]. Choi et al. showed that QDs (<5.5 nm) were able to be cleared from the body through the kidney and no accumulation of QDs was observed in the major organs of Sprague–Dawley (SD) male rats [26]. In our case, the  CdSe/ZnS QDs are remained the body because the hydrodynamic diameter of the  CdSe/ZnS QDs are greater than 6.5 nm. Thus, factors such as hydrodynamic size, surface coating and aggregation state of the QDs will play important roles in determining the ultimate fate of biodistribution and clearance of QDs [27, 28].

From animal studies, our data manifested that lymphocytes from QDs-treated mice (42 days) showed inhibited cell viability, changed proportions of total lymphocytes, enhanced release of TNF-α and IL-6, and inhibited transformation ability in response to LPS. It should be noted that in vitro exposure to QDs of lymphocytes from normal mice caused increased metabolic ability, which is opposite to the data obtained from lymphocytes of QDs-treated mice. It might result from the different exposure time of lymphocytes to QDs. The blood clearance profile revealed that the CdSe/ZnS QDs can be cleared by the blood quickly, and then QDs migrate to immune organs. CdSe/ZnS QDs were found to highly accumulate in the spleen within 42 days of evaluation period. The long-term exposures to QDs lead to cell viability damage to spleen lymphocytes.

In addition, our in vitro data showed that CdSe/ZnS QDs exposure led to immune dysfunction of macrophages, but our in vivo data revealed no obvious overt toxicity towards non-specific immunity of peritoneal macrophages in mice. The in vitro model reflects the direct bioactivity of nanoparticle-cell interaction and the in vivo system provides complex information about the response of a physiological system to a foreign substance. But since the exact exposure dose of CdSe/ZnS QDs between the in vitro assay and in vivo experiment is hard to compare, and the physiological process of in vitro and in vivo is not exactly the same, the results from in vitro assay and in vivo assay is not completely consistent. Similar with our founding, several studies have reported the inconsistent toxicity results from in vitro and in vivo assay. For example, Manna et al. reported in vitro toxicity of carbon nanotubes in human keratinocytes [29], whereas Schipper et al. found no significant toxicity in mice [30]. Similarly, Sayes et al. illustrated a lack of correlation when comparing the results between in vitro and in vivo toxicity assessments for fullerenes [31]. Compared to in vitro studies, in vivo animal model is a more preferable system to be used for the toxicological evaluation of nanoparticles because the biological aspects of the animal models are more relevant to human biology in many ways.

## Conclusions

Due to the ever-increasing application of QDs in many fields, the scale of nanotoxicology tests is expected to expand in the near future. The in vitro toxicity tests in combination with in vivo immunotoxicity in biological systems will play a pivotal role in assessing the toxicity of QDs and understanding the molecular mechanisms of nanotoxicity of QDs. Here, we report the potential toxicity of CdSe/ZnS QDs on immune cells from in vitro cell model and in vivo BALB/c mice for the first time. For in vitro studies, our results demonstrated that CdSe/ZnS QDs had toxic effects on macrophages and lymphocytes. At high doses, CdSe/ZnS QDs caused cytotoxicity towards macrophages by inducing ROS generation and apoptosis. In addition, CdSe/ZnS QDs exposure led to suppressive immune response of immune cells towards foreign stimuli and changed immune functionality. For in vivo studies, our data demonstrated that QDs injection by tail vein led to the accumulation of QDs in major immune organs, changed proportions of lymphocytes, and suppressive immune function of spleen-derived lymphocytes. In this regard, our results suggested that pre-exposure of CdSe/ZnS QDs might influence immune cell function and impair immune responses towards foreign stimuli, which in turn could result in decreased defense ability against infection and increased susceptibility of hosts to diseases.

## Additional file

10.1186/s12951-016-0162-4 Results from flow cytometry analysis of QDs uptake, ICP-MS analysis of Se element in major organs, the phagocytic assay of peritoneal macrophages, and MTT assay of macrophages treated with different types of QDs are presented.

## References

[1] Bruchez MJ, Moronne M, Gin P, Weiss S, Alivisatos AP (1998). Semiconductor nanocrystals as fluorescent biological labels. Science.

[2] Hauck TS, Anderson RE, Fischer HC, Newbigging S, Chan WCW (2010). In vivo Quantum-Dot Toxicity Assessment. Small.

[3] Liu J, Law W, Liu J, Hu R, Liu L, Zhu J, Chen H, Wang J, Hu Y, Ye L, Yong K (2013). Toxicity assessment of phospholipid micelle-encapsulated cadmium-based quantum dots using Kunming mice. RSC Adv.

[4] Qu G, Wang X, Wang Z, Liu S, Jiang G (2013). Cytotoxicity of quantum dots and graphene oxide to erythroid cells and macrophages. Nanoscale Res Lett.

[5] Nguyen KC, Rippstein P, Tayabali AF, Willmore WG (2015). Mitochondrial Toxicity of Cadmium Telluride Quantum Dot Nanoparticles in Mammalian Hepatocytes. Toxicol Sci.

[6] Pathakoti K, Hwang HM, Xu H, Aguilar ZP, Wang A (2013). In vitro cytotoxicity of CdSe/ZnS quantum dots with different surface coatings to human keratinocytes HaCaT cells. J Environ Sci (China).

[7] Chen N, He Y, Su Y, Li X, Huang Q, Wang H, Zhang X, Tai R, Fan C (2012). The cytotoxicity of cadmium-based quantum dots. Biomaterials.

[8] Mahto SK, Park C, Yoon TH, Rhee SW (2010). Assessment of cytocompatibility of surface-modified CdSe/ZnSe quantum dots for BALB/3T3 fibroblast cells. Toxicol In Vitro.

[9] Nguyen KC, Seligy VL, Tayabali AF (2013). Cadmium telluride quantum dot nanoparticle cytotoxicity and effects on model immune responses to Pseudomonas aeruginosa. Nanotoxicology.

[10] Lin G, Ouyang Q, Hu R, Ding Z, Tian J, Yin F, Xu G, Chen Q, Wang X, Yong K (2015). In vivo toxicity assessment of non-cadmium quantum dots in BALB/c mice. Nanomed Nanotechnol Biol Med.

[11] Ye L, Yong K, Liu L, Roy I, Hu R, Zhu J, Cai H, Law W, Liu J, Wang K (2012). Others: a pilot study in non-human primates shows no adverse response to intravenous injection of quantum dots. Nat Nanotechnol.

[12] Lin G, Ding Z, Hu R, Wang X, Chen Q, Zhu X, Liu K, Liang J, Lu F, Lei D (2014). Cytotoxicity and immune response of CdSe/ZnS quantum dots towards a murine macrophage cell line. RSC Adv.

[13] Clift MJD, Varet J, Hankin SM, Brownlee B, Davidson AM, Brandenberger C, Rothen-Rutishauser B, Brown DM, Stone V (2011). Quantum dot cytotoxicity in vitro An investigation into the cytotoxic effects of a series of different surface chemistries and their core/shell materials. Nanotoxicology.

[14] Lin G, Wang X, Yi W, Zhang C, Xu G, Zhu X, Cai Z, Liu Y, Diao Y, Lin MC, Jin G (2015). A conjugate of octamer-binding transcription factor 4 and toll-like receptor 7 agonist prevents the growth and metastasis of testis embryonic carcinoma. J Transl Med.

[15] Rietschel ET, Kirikae T, Schade FU, Mamat U, Schmidt G, Loppnow H, Ulmer AJ, Zahringer U, Seydel U, Di Padova F, Et A (1994). Bacterial endotoxin: molecular relationships of structure to activity and function. FASEB J.

[16] Weiner GJ, Liu HM, Wooldridge JE, Dahle CE, Krieg AM (1997). Immunostimulatory oligodeoxynucleotides containing the CpG motif are effective as immune adjuvants in tumor antigen immunization. Proc Natl Acad Sci USA.

[17] Hardman R (2006). A toxicologic review of quantum dots: toxicity depends on physicochemical and environmental factors. Environ Health Perspect.

[18] Yuanyuan S, Yao H, Haoting L, Liman S, Qingnuan L, Wenxin L, Lianhui W, Pingping S, Qing H, Chunhai F (2009). The cytotoxicity of cadmium based, aqueous phase—synthesized, quantum dots and its modulation by surface coating. Biomaterials.

[19] Shiohara A, Hoshino A, Hanaki K, Suzuki K, Yamamoto K (2004). On the cyto-toxicity caused by quantum dots. Microbiol Immunol.

[20] Lee HM, Shin DM, Song HM, Yuk JM, Lee ZW, Lee SH, Hwang SM, Kim JM, Lee CS, Jo EK (2009). Nanoparticles up-regulate tumor necrosis factor-alpha and CXCL8 via reactive oxygen species and mitogen-activated protein kinase activation. Toxicol Appl Pharmacol.

[21] Zhang Z, Berg A, Levanon H, Fessenden RW, Meisel D (2003). On the interactions of free radicals with gold nanoparticles. J Am Chem Soc.

[22] Manke A, Wang L, Rojanasakul Y (2013). Mechanisms of nanoparticle-induced oxidative stress and toxicity. Biomed Res Int.

[23] Hsin YH, Chen CF, Huang S, Shih TS, Lai PS, Chueh PJ (2008). The apoptotic effect of nanosilver is mediated by a ROS- and JNK-dependent mechanism involving the mitochondrial pathway in NIH3T3 cells. Toxicol Lett.

[24] Takeda K, Akira S (2004). TLR signaling pathways. Semin Immunol.

[25] Mullick CS, Lalwani G, Zhang K, Yang JY, Neville K, Sitharaman B (2013). Cell specific cytotoxicity and uptake of graphene nanoribbons. Biomaterials.

[26] Choi HS, Liu W, Misra P, Tanaka E, Zimmer JP, Itty IB, Bawendi MG, Frangioni JV (2007). Renal clearance of quantum dots. Nat Biotechnol.

[27] Su Y, Peng F, Jiang Z, Zhong Y, Lu Y, Jiang X, Huang Q, Fan C, Lee ST, He Y (2011). In vivo distribution, pharmacokinetics, and toxicity of aqueous synthesized cadmium-containing quantum dots. Biomaterials.

[28] Tang Y, Han S, Liu H, Chen X, Huang L, Li X, Zhang J (2013). The role of surface chemistry in determining in vivo biodistribution and toxicity of CdSe/ZnS core-shell quantum dots. Biomaterials.

[29] Manna SK, Sarkar S, Barr J, Wise K, Barrera EV, Jejelowo O, Rice-Ficht AC, Ramesh GT (2005). Single-walled carbon nanotube induces oxidative stress and activates nuclear transcription factor-kappaB in human keratinocytes. Nano Lett.

[30] Schipper ML, Nakayama-Ratchford N, Davis CR, Kam NW, Chu P, Liu Z, Sun X, Dai H, Gambhir SS (2008). A pilot toxicology study of single-walled carbon nanotubes in a small sample of mice. Nat Nanotechnol.

[31] Sayes CM, Marchione AA, Reed KL, Warheit DB (2007). Comparative pulmonary toxicity assessments of C60 water suspensions in rats: few differences in fullerene toxicity in vivo in contrast to in vitro profiles. Nano Lett.

